# Serum C-X-C motif chemokine 13 is elevated in early and established rheumatoid arthritis and correlates with rheumatoid factor levels

**DOI:** 10.1186/ar4552

**Published:** 2014-04-25

**Authors:** Jonathan D Jones, B JoNell Hamilton, Gregory J Challener, Artur J de Brum-Fernandes, Pierre Cossette, Patrick Liang, Ariel Masetto, Henri A Ménard, Nathalie Carrier, David L Boyle, Sanna Rosengren, Gilles Boire, William F C Rigby

**Affiliations:** 1 Division of Rheumatology, Geisel School of Medicine at Dartmouth College, One Medical Center Dr., Lebanon, NH 03756, USA; 2 Department of Microbiology and Immunology, Geisel School of Medicine at Dartmouth College, One Medical Center Dr., Lebanon, NH 03756, USA; 3 Division of Rheumatology, Sherbrooke University Hospital, 2500 University Boulevard, Sherbrooke, QC J1K 2R1, Canada; 4 Department of Medicine, Sherbrooke University Hospital, 2500 University Boulevard, Sherbrooke, QC J1K 2R1, Canada; 5 Research Institute of the McGill University Health Center, 2155 Guy St Montreal, QC H3H 2R9, Canada; 6 Division of Rheumatology, University of California San Diego School of Medicine, 200 W Arbor Dr, La Jolla, CA 92103, USA; 7 Halozyme Therapeutics, 11388 Sorrento Valley Road, San Diego, CA 92121, USA

## Abstract

**Introduction:**

We hypothesized that serum levels of C-X-C motif chemokine 13 (CXCL13), a B-cell chemokine, would delineate a subset of rheumatoid arthritis (RA) patients characterized by increased humoral immunity.

**Methods:**

Serum from patients with established RA (the Dartmouth RA Cohort) was analyzed for CXCL13, rheumatoid factor (RF) levels, anticitrullinated peptide/protein antibody (ACPA) and total immunoglobulin G (IgG); other parameters were obtained by chart review. A confirmatory analysis was performed using samples from the Sherbrooke Early Undifferentiated PolyArthritis (EUPA) Cohort. The Wilcoxon rank-sum test, a *t*-test and Spearman’s correlation analysis were utilized to determine relationships between variables.

**Results:**

In both the Dartmouth and Sherbrooke cohorts, CXCL13 levels were selectively increased in seropositive relative to seronegative RA patients (*P* = 0.0002 and *P* < 0.0001 for the respective cohorts), with a strong correlation to both immunoglobulin M (IgM) and IgA RF levels (*P* < 0.0001). There was a weaker relationship to ACPA titers (*P* = 0.03 and *P* = 0.006, respectively) and total IgG (*P* = 0.02 and *P* = 0.14, respectively). No relationship was seen with regard to age, sex, shared epitope status or inclusion high-sensitivity C-reactive protein (hsCRP) in either cohort or regarding the presence of baseline erosions in the Sherbrooke Cohort, whereas a modest relationship with Disease Activity Score in 28 joints CRP (DAS28-CRP) was seen in the Dartmouth cohort but not the Sherbrooke cohort.

**Conclusion:**

Using both established and early RA cohorts, marked elevations of serum CXCL13 levels resided nearly completely within the seropositive population. CXCL13 levels exhibited a strong relationship with RF, whereas the association with clinical parameters (age, sex, DAS28-CRP and erosions) or other serologic markers (ACPA and IgG) was either much weaker or absent. Elevated serum CXCL13 levels may identify a subset of seropositive RA patients whose disease is shaped by or responsive to RF production.

## Introduction

Seropositive rheumatoid arthritis (RA) is an inflammatory disease characterized by autoantibodies (immunoglobulin G (IgG) anticitrullinated peptide/protein antibodies (ACPAs) and rheumatoid factor (RF)). These autoantibodies can appear years before the onset of clinical disease and are strongly linked to the human leukocyte antigen major histocompatibility complex class II DR β1 (HLA-DRB1) alleles containing the shared epitope [1]. The presence of IgG ACPAs and IgA-RF indicates that antibody heavy-chain class-switching has occurred, which is typically associated with T-cell–dependent B-cell maturation and differentiation [2,3].

An important element of T-cell–dependent B cell maturation and differentiation is the formation of lymphoid follicles and germinal centers. Murine studies indicate the interaction of the C-X-C motif chemokine 13 (CXCL13) with C-X-C chemokine receptor type 5 (CXCR5) promotes this process through the recruitment of naïve B cells and follicular T cells to the lymphoid follicle [4-6]. Thus, it seems reasonable to posit that CXCL13 plays a role in the development of both IgG ACPAs and IgA-RF prior to the development of clinical signs and symptoms.

In addition to the development of autoantibodies in the preclinical phase, CXCL13 has been associated with synovial inflammation in RA. A series of observations has established its production by multiple cell types in rheumatoid synovium, frequently in association with the formation of lymphoid follicular structures, including synovial T cells (but not T follicular cells) [7], monocytes/macrophages [8] and follicular dendritic cells, endothelial cells and synovial fibroblasts [9].

In addition to its synovial production in RA, elevated serum levels of CXCL13 have been observed and were reported to be 1.7× higher in one small study of patients with active relative to quiescent disease [10]. Rosengren *et al*. subsequently established the presence of a strong relationship between synovial CXCL13 mRNA expression and serum CXCL13 level in a cohort of patients with long-standing RA [11]. Thus, synovial production appears to account for increased serum CXCL13 levels rather than serum CXCL13 elevation arising from a systemic reaction to joint inflammation.

Given those data, we hypothesized that serum CXCL13 levels would reflect the impact of CXCL13 on synovial inflammation and the shaping of the clinical and serologic phenotype. We specifically wished to determine if CXCL13 levels identify a subset of RA patients, perhaps indicating a greater role of humoral immunity in disease pathogenesis. We undertook a cross-sectional analysis of circulating serum CXCL13 levels in RA patients followed at the Dartmouth-Hitchcock Medical Center. In this cohort, we observed that CXCL13 expression was much higher in seropositive than seronegative RA patients. In addition, we observed that this relationship correlated most strongly with RF and not with ACPA. Subsequently, we saw identical relationships in an early RA cohort. We performed genetic, serologic and clinical analyses, which indicated that serum CXCL13 levels may identify a novel (and abundant) subpopulation of seropositive RA. Additional studies are required to assess the utility of this biomarker.

## Methods

### Patient samples

The Dartmouth RA Cohort consists of patients recruited from the Dartmouth-Hitchcock Medical Center Rheumatology Clinic (Lebanon, NH, USA) who have established RA defined according to the American College of Rheumatology (ACR) 1987 revised criteria [12]. This cohort represents a patient population with established RA whose disease duration extends, in some cases, longer than 20 years (Table 1). Approval for this study was obtained from the Committee for the Protection of Human Subjects at Dartmouth College, and the patients provided their informed, signed consent to participate. Age and sex, disease duration, medication history, smoking status, seropositivity (determined by clinical laboratory determination of RF >14 IU/ml using immunoturbidimetric measurement (Roche Diagnostics, Indianapolis, IN, USA) and/or anti–cyclic citrullinated peptide 2 (anti-CCP2) >5.0 U/ml by enzyme-linked immunosorbent assay (ELISA) (DiaSorin, Saluggia, Italy)) and high-sensitivity C-reactive protein (hsCRP) levels were recorded, and serum and DNA were collected. In some cases, Disease Activity Score in 28 joints CRP (DAS28-CRP) and Clinical Disease Activity Index (CDAI) scores were available from the clinical charts.

**Table 1:** **Patients in the Dartmouth RA Cohort**^**a**^

**Patient demographics (*N *** **= 193)**	**Seronegative**	**Seropositive**
Number of patients (%)	30 (16%)	163 (84%)
Average age, yr (range)	55.6 (29 to 72)	57.9 (19 to 92)
Females, *n* (%)	24 (80%)	115 (71%)
RA duration <2 yr	10 (33%)	41 (25%)
SE status, *n*	14	116
SE alleles = 0	7	22
SE alleles = 1	7	56
SE alleles = 2	0	38
C4 status, *n*	14	111
C4 copies <4	9	30
C4 copies ≥4	5	81
Medication history, *n*		
No DMARDs	9	36
Nonbiologic DMARDs	10	54
TNF inhibitors	4	42
Other biologics	7^b^	31^c^

^a^C4, Complement 4; DMARD, Disease-modifying antirheumatic drug; RA, Rheumatoid arthritis; SE, Shared epitope; TNF, Tumor necrosis factor. ^b^Abatacept (*n* = 1) and rituximab (*n* = 6). ^c^Abatacept (*n* = 2), rituximab (*n* = 27), tocilizumab (*n* = 1) and tofacitinib (*n* = 1).

The confirmatory cohort consists of a subset of the patients recruited from Sherbrooke, QC, Canada, as part of the longitudinal Early Undifferentiated Polyarthritis (EUPA) Cohort. This cohort varies from the Dartmouth RA Cohort in that it represents an early arthritis population, contains a greater proportion of seronegative patients and has predominantly patients who were DMARD- and corticosteroid-naïve at the time of inclusion. Cohort inclusion criteria included age ≥18 years, disease duration between 1 and 12 months and swollen joint count of three or more. In the subset reported herein, all RA patients fulfilled the 1987 ACR revised criteria. Seropositivity was defined as both an RF titer ≥40 IU/ml measured using RapiTex RF (Dade Behring, Deerfield, IL, USA) and anti-CCP2 >20 U/ml using QUANTA Lite (Inova Diagnostics, San Diego, CA, USA), present concurrently at least once. Seronegativity was defined as negative RF and anti-CCP2 at all the visits. This subset was chosen randomly from among the Sherbrooke EUPA Cohort, with samples matched only for serostatus. The patients provided their signed, informed consent to participate, and study approval was obtained from the Sherbrooke University Hospital Centre Institutional Review Board. Information gathered at enrollment included demographic details such as age, sex and time since onset of arthritis, as well as clinical details such as DAS28-CRP score, radiologic Sharp score and RF and ACPA status (Table 2).

**Table 2:** **Recent-onset rheumatoid arthritis patients in the Sherbrooke Early Undifferentiated PolyArthritis Cohort**^**a**^

**Patient demographics (*N *** **= 339)**	**Seronegative**	**Seropositive**
Number of patients (%)	166 (49%)	173 (51%)
Average age, yr (range)	54.2 (21 to 92)	65.6 (19 to 87)
Females, *n* (%)	107 (64)	105 (61)
Symptom duration, yr	0.44	0.45
SE status, *n*	120	132
SE alleles = 0	75	43
SE alleles = 1	44	59
SE alleles = 2	1	30
Mean DAS28-CRP (±SD)	5.10 (1.47)	5.04 (1.44)
Mean CRP mg/L (±SD)	24.4 (33.9)	26.6 (35.6)
Mean Sharp score erosions (±SD)	2.9 (6.1)	3.8 (7.0)
Mean Sharp score narrowing (±SD)	3.4 (6.1)	2.2 (4.1)
Tender joints	10.4	9.8
Swollen joints	10.5	10.2
Patient VAS score (100-mm scale)	54.2	53.9

^a^CRP, C-reactive protein; DAS28-CRP, Disease Activity Score in 28 joints C-reactive protein; SE, Shared epitope; VAS, Visual Analogue Scale. Seronegative vs seropositive analysis: Symptom duration, *P* = 0.85; DAS28-CRP, *P* = 0.72; CRP, *P* = 0.56; Sharp score erosions, *P* = 0.28; Sharp score narrowing, *P* = 0.09.

### Serum analysis

Patient serum was stored at −80°C (Dartmouth RA Cohort) or −20°C (Sherbrooke EUPA Cohort) until analysis. CXCL13 levels were measured by ELISA according to the manufacturer’s instructions (Human CXCL13/BLC/BCA-1 DuoSet; R&D Systems, Minneapolis, MN, USA). Several samples were kept and repetitively measured over the course of 3 weeks at 4°C, with no change in CXCL13 levels compared to freshly thawed serum. Serum samples were diluted 1:100, but the ELISA was repeated at a serum dilution of 1:10 in cases where low values were obtained. In six RF-positive patient samples, HeteroBlock reagent (Omega Biologicals, Bozeman, MT, USA) was titrated into the assay to eradicate the potential for false-positive CXCL13 results due to the heterophilic activity of RF. We noticed that our range of CXCL13 levels was greater than levels reported elsewhere in the literature, so we confirmed the results in the Dartmouth RA Cohort using a premade ELISA kit from the same company (R&D Systems), which showed minimal variation from the original values we obtained (r = 0.95).

Total levels of IgG were measured by ELISA (Immunology Consultants Laboratory, Portland, OR, USA), as were IgM RF levels (TheraTest Laboratories, Lombard, IL, USA) and IgA RF levels (Inova Diagnostics). In the Dartmouth RA Cohort, we additionally measured serum levels of IgG ACPA using a human QUANTA Lite CCP3 IgG ELISA kit (Inova Diagnostics). All analyses were done on the same sample or with samples from an aliquot identical to the sample used to measure CXCL13. The one exception was the measurement of anti-CCP2 levels (Inova Diagnostics/EUROIMMUN US, Morris Plains, NJ, USA) in the Sherbrooke EUPA Cohort, as it was measured either simultaneously with or within a few weeks after the serum collection used for the CXCL13 assay.

### DNA analysis

In the Dartmouth RA Cohort, HLA-DRB1 status (shared epitope) was obtained previously [13] through the American Red Cross Penn-Jersey Blood Services Region. Complement 4 (*C4)* allele copy number was also obtained as described previously [13] by Southern blot analysis, with confirmation by RT-PCR. HLA-DRB1 typing in the Sherbrooke EUPA Cohort was determined using sequence-specific primer PCR techniques as previously described [14].

### Statistical analysis

Statistical analysis was performed using STATA software version 12.1 (StataCorp, College Station, TX, USA). CXCL13, hsCRP and IgA RF levels were log-transformed because of the wide range and non-normal distribution of the data. Comparisons of two means were carried out by independent Student’s *t*-test or by Wilcoxon rank-sum test for non-normal distributions. Pearson correlation or Spearman correlation (in non-normal distributions) were used for analysis of log-transformed CXCL13 and other measures, such as IgM RF, ACPA, total IgG, age, hsCRP, DAS28-CRP, CDAI and erosions. For additional analysis of CXCL13 relationships to RF and ACPA, CXCL13 values from seropositive patients were divided into tertiles. The lower and upper cutoffs for the Dartmouth RA Cohort were 160 and 400 pg/ml, respectively. For the Sherbrooke EUPA Cohort, the lower and upper cutoffs were 150 and 1100 pg/ml, respectively. Because of clear overlap of RF values of the lower two CXCL13 tertiles, these values were combined for comparison to the highest tertile. Two-tailed *P* values <0.05 were considered significant.

## Results

### CXCL13 is elevated in seropositive rheumatoid arthritis patients and correlates with immunoglobulin M rheumatoid factor

**Figure 1:**
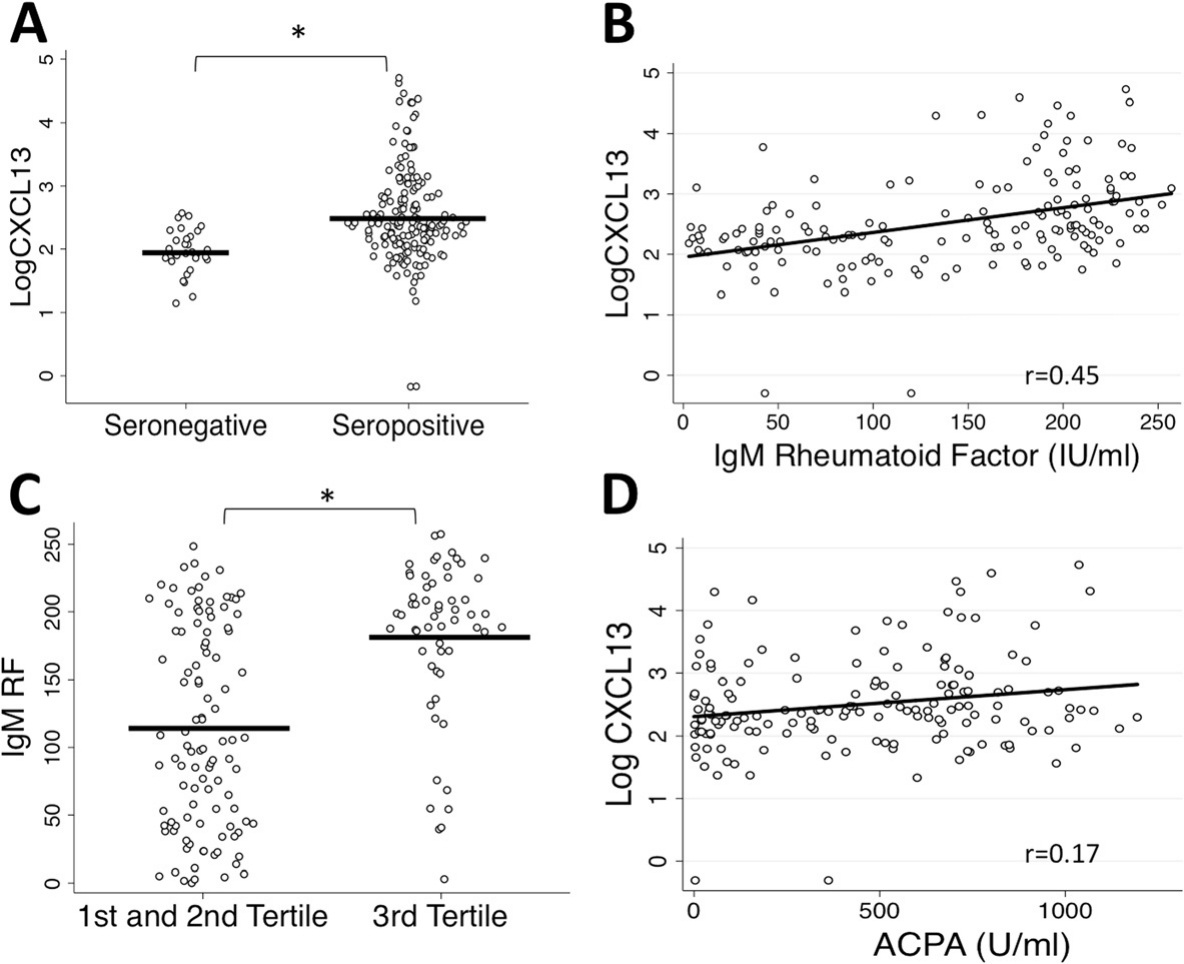
**Scatterplots illustrate strong correlation of serum CXCL13 with seropositivity and immunoglobulin M rheumatoid factor in the Dartmouth RA Cohort. (A)** Log-transformed C-X-C motif chemokine 13 (CXCL13) values are higher in seropositive compared to seronegative rheumatoid arthritis (RA) patients by *t*-test (seropositive (*n* = 163), geometric mean (95% CI) = 331.1 pg/ml (250.0 to 430.5); seronegative (*n* = 30), geometric mean (95% CI) 93.3 pg/ml (71.3 to 123.9); *P* = 0.0002). **(B)** The log-transformed CXCL13 values of seropositive patients increase with higher rheumatoid factor (RF) levels when evaluated by Spearman correlation (*P* < 0.0001). **(C)** Tertile analysis of seropositive patients comparing the highest CXCL13 tertile (third tertile) to the first and second tertiles by Wilcoxon rank-sum test identifies a strong relationship with immunoglobulin M (IgM) RF (third-tertile IgM RF: mean = 182 ± 59 IU/ml; first- and second-tertile IgM RF: mean = 113 ± 74 IU/ml; *P* < 0.0001). **(D)** CXCL13 has a weaker relationship to IgG anticitrullinated peptide/protein antibody (ACPA) (*P* = 0.03). *P < 0.05. Diagonal lines represent line of best fit.

The Dartmouth RA Cohort (*N* = 193) represents an established RA cohort with a variation in disease duration from <1 year to >20 years (Table 1). We first analyzed serum CXCL13 levels in seronegative patients in relation to seropositive patients, as determined by the clinical laboratory data and chart history. Owing to the range of CXCL13 levels obtained (0 to >53,000 pg/ml) and the non-normal distributions, the data were log-transformed (log CXCL13). We identified a significant elevation in log CXCL13 levels in seropositive patients (by independent Student’s *t*-test), with a geometric mean values (95% CI) of 93 pg/ml in seronegatives (71.3 to 123.9) and 331 pg/ml in seropositives (250.0 to 430.5) (*P* = 0.0002) (Figure 1A). The addition of HeteroBlock did not alter the results, thus confirming that these findings were not due to the presence of RF in the seropositive sera (data not shown).

Serum IgM RF and IgG ACPA levels were measured in the seropositive patients and evaluated by Spearman correlation in relation to log CXCL13 levels. We found a highly significant relationship to IgM RF (*r* = 0.45, *P* < 0.0001) (Figure 1B), with a much weaker relationship to IgG ACPA (*r* = 0.17, *P* = 0.03) (Figure 1D). Of the 163 seropositive patients, 7 patients (4.3%) were positive for IgG ACPA but negative for IgM RF, and 8 patients (4.9%) were negative for IgG ACPA but positive for IgM RF. Evaluation of the CXCL13 values of these single-positive samples did not differ from the remaining double-positive samples (data not shown). Tertile analysis of CXCL13 values confirmed that IgM RF was higher in the third than in the first and second tertiles (mean = 182 ± 59 vs 113 ± 74 U/ml; *P* < 0.0001) (Figure 1C) but that IgG ACPA was not (mean = 511 ± 319 vs. 419 ± 339 U/ml; *P* = 0.09) (data not shown), as determined by Wilcoxon rank-sum test. Evaluation of log CXCL13 levels in relation to total IgG levels showed a weak but statistically significant relationship (*r* = 0.18, *P* = 0.02) (data not shown).

We examined the potential relationship of log CXCL13 levels to genetic markers (shared epitope status and complement *C4B* gene copy number deficiency) associated with autoantibody positivity in RA [13,15]. We saw no relationship between log CXCL13 levels in seropositive RA patients (*n* = 115 patients with HLA-DRB1 determination) in the presence or absence of the shared epitope (*P* = 0.91) (data not shown). Similarly, no relationship was seen (*n* = 111 patients with C4 (please italicize) gene copy number determination) with *C4B* gene copy number deficiency (*P* = 0.69), *C4A* deficiency (*P* = 0.56), or total *C4* deficiency, defined as *C4* gene copy number <4 (*P* = 0.35) (data not shown).

### CXCL13 relationships to other serologic and clinical features

**Figure 2:**
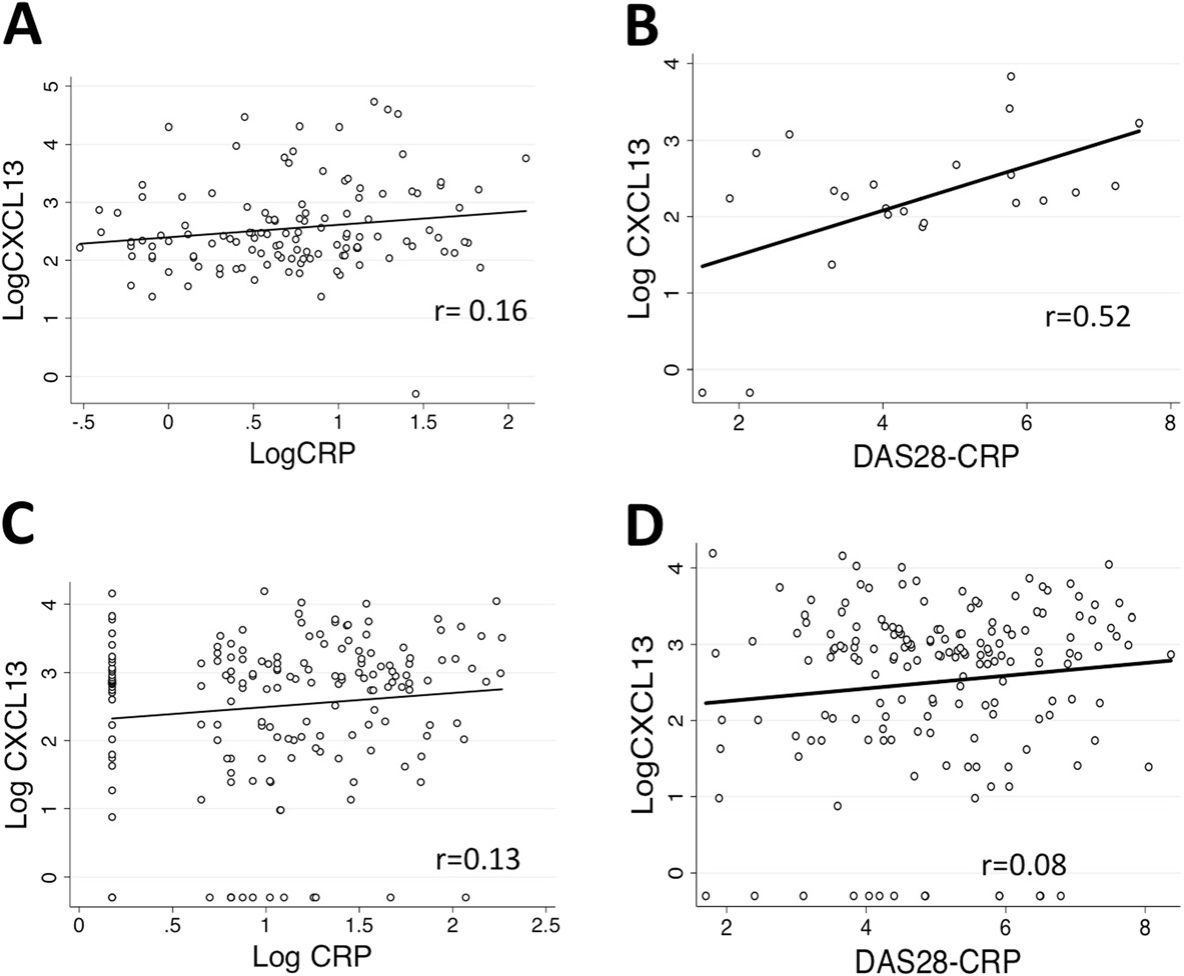
**Scatterplots show minimal or no relationship of serum CXCL13 to high-sensitivity C-reactive protein or disease activity score in 28 joints C-reactive protein in the seropositive groups of the Dartmouth RA Cohort and the Sherbrooke Early Undifferentiated PolyArthritis Cohort. (A)** In the Dartmouth RA Cohort, correlation analysis of log-transformed C-X-C motif chemokine 13 (LogCXCL13) and high-sensitivity C-reactive protein (LogCRP) does not show a relationship (*n* = 123, *P* = 0.07). **(B)** In the Dartmouth RA Cohort, correlation analysis of CXCL13 has a modest relationship with Disease Activity Score in 28 joints CRP (DAS28-CRP) (*n* = 23, *P* = 0.01). **(C)** In the recent-onset rheumatoid arthritis (RA) patients from the Sherbrooke Early Undifferentiated PolyArthritis (EUPA) Cohort, no relationship is identified between LogCXCL13 and LogCRP (*n* = 173, *P* = 0.08). **(D)** In the recent-onset RA patients from the Sherbrooke EUPA Cohort, no relationship is identified between LogCXCL13 and DAS28-CRP (*n* = 170, *P* = 0.28). Diagonal lines represent lines of best fit.

Within this cross-sectional analysis, we examined the relationship of CXCL13 levels in relation to laboratory-reported hsCRP levels at the time of sample collection (*n* = 123 seropositive patients) and various clinical assessments of disease activity, namely, DAS28-CRP and CDAI. Log-transformed hsCRP (log CRP) and log CXCL13 showed only a trend toward significance (*P* = 0.07) (Figure 2A). Simultaneous measures of DAS28-CRP and CDAI on 23 and 22 seropositive patients, respectively, were available. The DAS28-CRP association was shown to be significant (r = 0.52, *P* = 0.01) (Figure 2B), whereas the CDAI showed only a trend toward a relationship to log CXCL13 (*r* = 0.38, *P* = 0.08) (data not shown). Further analyses included comparing log CXCL13 to age and sex, but the results were unremarkable (*P* = 0.28 and *P* = 0.34 respectively) (data not shown). Additionally, we found no relationship of log CXCL13 to smoking status as defined by never-smokers, past smokers and current smokers (*P* = 0.47).

### Relationships between CXCL13 and antibody levels in an early rheumatoid arthritis cohort

**Figure 3:**
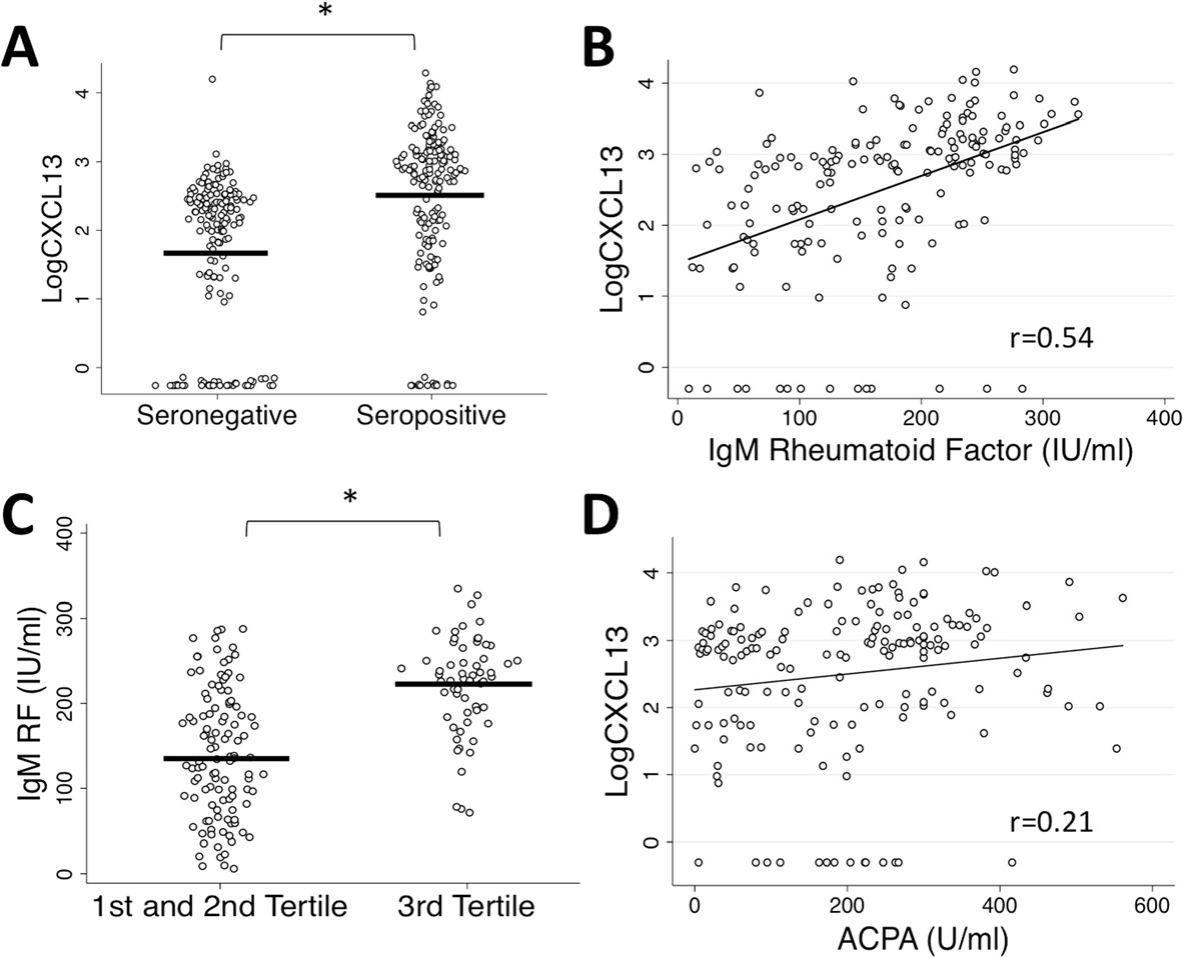
**Scatterplots demonstrate serum CXCL13 strongly correlates with seropositive rheumatoid arthritis and immunoglobulin M rheumatoid factor levels in the Sherbrooke Early Undifferentiated PolyArthritis Cohort, but has a weaker relationship to anticitrullinated peptide/protein antibody levels. (A)** Log-transformed C-X-C motif chemokine 13 (LogCXCL13) is higher in seropositive than in seronegative RA patients as determined by *t*-test (seropositive: *n* = 173, geometric mean (95% CI) = 323.6 pg/ml (223.9 to 477.5); seronegative: *n* = 166, geometric mean (95% CI) = 50.1 pg/ml (35.0 to 78.0); *P* < 0.0001). **(B)** As with the Dartmouth RA Cohort, a strong relationship is seen with LogCXCL13 and immunoglobulin M rheumatoid factor (IgM RF) in seropositive samples, whether measured by Spearman correlation (*P* < 0.0001) **(C)** or by CXCL13 tertile analysis (third-tertile mean RF = 223 ± 57 IU/ml, first- and second-tertile mean RF = 141 ± 75 IU/ml; *P* < 0.0001). **(D)** A significant relationship is found between CXCL13 and anticitrullinated peptide/protein antibody (ACPA) by Spearman correlation (*P* = 0.006). *P < 0.05. Diagonal lines represent lines of best fit.

We compared our findings in an established RA cohort to that of a well-characterized early RA cohort (the Sherbrooke EUPA Cohort) to address any potential confounding by current or past therapy. This cohort consisted of a nearly equal number of seronegative and seropositive patients (*n* = 166 and 173, respectively) with an average disease duration of approximately 5 months (Table 2). As with established RA, a strong relationship with log CXCL13 levels and seropositivity had already been seen at the inclusion visit (*P* < 0.0001) (Figure 3A) with a geometric mean (95% CI) of 50.1 pg/ml (35.0 to 78.0) in seronegatives and 323.6 pg/ml (223.9 to 477.5) in seropositives. Similarly, we observed a strong relationship in the seropositive patients when log CXCL13 levels were evaluated by Spearman correlation analysis against IgM RF levels (*r* = 0.54, *P* < 0.0001) (Figure 3B) as well as by tertile analysis (*P* < 0.0001) (Figure 3C). In comparison to the patients with established RA in the Dartmouth RA Cohort, the recent-onset RA patients showed a stronger relationship between CXCL13 and IgG ACPA with *P* = 0.006 (*r* = 0.21) (Figure 3D) and *P* = 0.02 by tertile analysis (data not shown), respectively, but no relationship between serum IgG and CXCL13 level was observed (*r* = 0.11, *P* = 0.14) (data not shown). As we observed with the Dartmouth RA Cohort, we found no relationship with shared epitope status in the seropositives (*n* = 132; *P* = 0.38) (data not shown) or with smoking status (*P* = 0.62). Additionally, when we combined the data set from both cohorts, we continued to find no relationship with either shared epitope or smoking status (Additional file 1).

### Relationships between CXCL13 and disease activity measures in an early rheumatoid arthritis cohort

The recent-onset RA patients drawn from the Sherbrooke EUPA Cohort showed no association between log CXCL13 serum levels and either age or sex (*P* = 0.77 and *P* = 0.43 respectively) (data not shown). A correlation between log CXCL13 and log CRP values were identified when seropositive and seronegative patients were combined (*n* = 339; *r* = 0.14, *P* = 0.01) (data not shown). This association dissipated when evaluated only in the seropositives (*n* = 173; *r* = 0.13, *P* = 0.08) (Figure 2C). A similar relationship of serum CXCL13 to DAS28-CRP levels was shown in all patients (*n* = 335; *r* = 0.12, *P* = 0.02), but this association also went away when the analysis was limited to seropositives (*n* = 170; *r* = 0.08, *P* = 0.28) (Figure 2D). There was no relationship between log CXCL13 levels and the presence of erosions at baseline in seropositive patients, regardless of whether it was evaluated by Spearman correlation according to number of erosions (*n* = 123; *r* = −0.12, *P* = 0.17) (data not shown) or by *t*-test to compare the presence or absence of erosions (0 vs ≥1 erosions, *P* = 0.34; <5 vs ≥5 erosions; *P* = 0.95).

### Relationships between serum CXCL13 and immunoglobulin A rheumatoid factor

Despite a strong relationship of serum CXCL13 with IgM RF seropositivity, both established and recent-onset RA cohorts exhibited weaker correlations between CXCL13 and serum IgG and IgG ACPA levels. These data suggest that elevated serum CXCL13 levels might correspond to a process in which non-class-switched B cells producing IgM RF were promoted independently of follicle and germinal center formation that leads to immunoglobulin heavy-chain class switching and IgG ACPA. To begin to address this model, we analyzed the relationship between serum CXCL13 levels and IgA RF.

**Figure 4:**
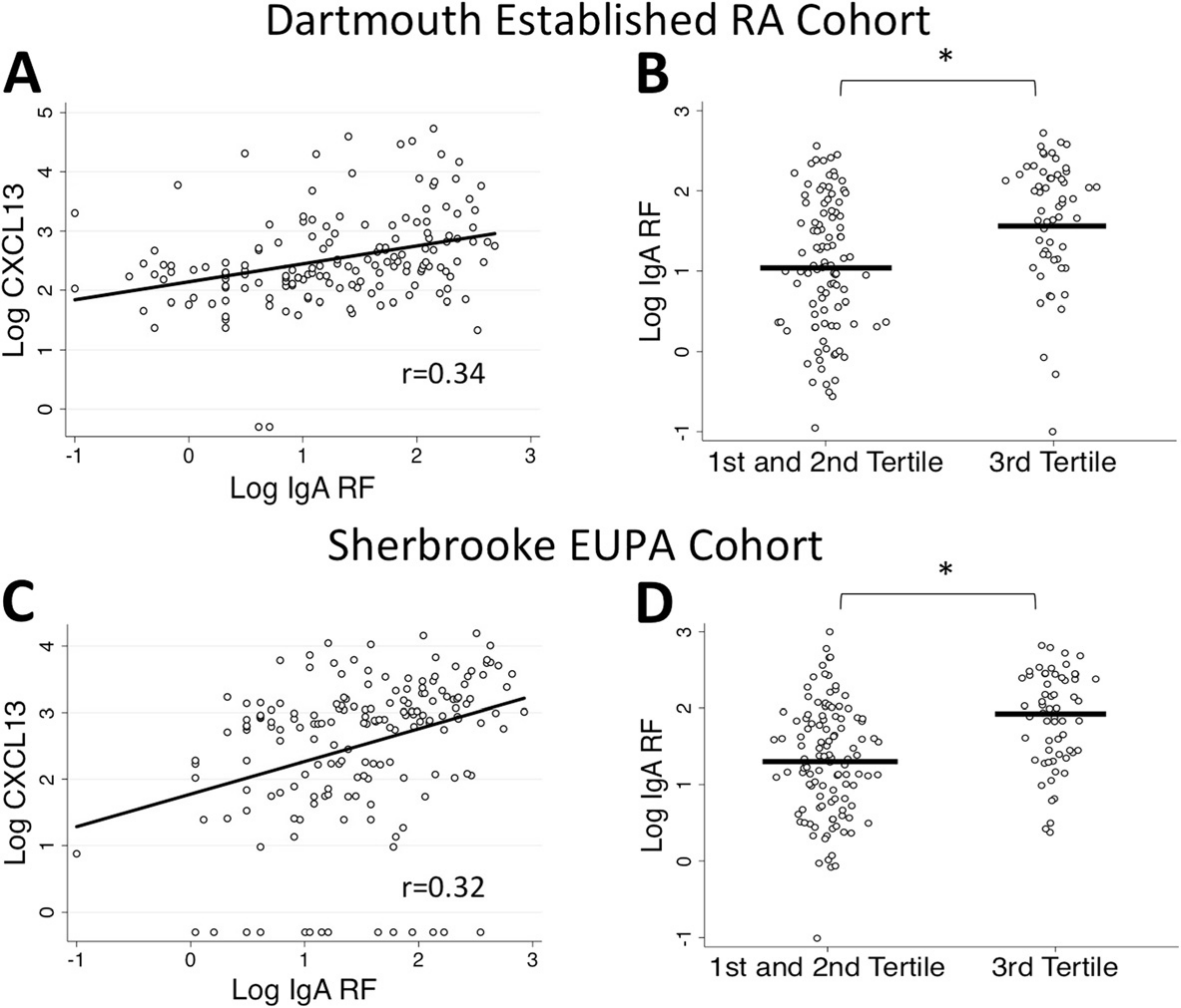
**Scatterplots illustrate a strong relationship of serum CXCL13 with immunoglobulin A rheumatoid factor in seropositive rheumatoid arthritis patients in both the Dartmouth RA Cohort (established RA) and recent-onset rheumatoid arthritis patients from the Sherbrooke Early Undifferentiated PolyArthritis Cohort. (A)** Pearson correlation analysis of log-transformed C-X-C motif chemokine 13 (LogCXCL13) values and log-transformed immunoglobulin A rheumatoid factor (Log IgA RF) in the Dartmouth RA Cohort shows a strong association (*P* < 0.0001). **(B)** CXCL13 tertile analysis confirms this relationship (third-tertile IgA RF: geometric mean (95% CI) = 45.0 IU/ml (28.0 to 72.3), first- and second-tertile IgA RF: geometric mean (95% CI) = 11.2 IU/ml (7.6 to 16.3); *P* < 0.0001). **(C)** In the Sherbrooke Early Undifferentiated PolyArthritis Cohort, the relationship between LogCXCL13 and Log IgA RF persists, whether measured by correlation (*P* < 0.0001) **(D)** or by CXCL13 tertile analysis (third-tertile IgA RF: geometric mean (95% CI) = 74.1 IU/ml (51.4 to 106.7), first- and second-tertile IgA RF: geometric mean (95% CI) = 20.4 IU/ml (15.1 to 27.5); *P* < 0.0001). *P < 0.05. Diagonal lines represent lines of best fit.

Analysis of log-transformed CXCL13 and log-transformed IgA RF in the seropositive patients of both cohorts were strongly correlated (*P* < 0.0001) (Figure 4A and 4C). When evaluated by CXCL13 tertile analysis, the highest tertile had much higher IgA RF values than the first and second tertiles in the Dartmouth RA Cohort (third-tertile geometric mean (95% CI) 45.0 IU/ml (28.0 to 72.3), first- and second-tertile geometric mean (95% CI) 11.2 IU/ml (7.6 to 16.3); *P* < 0.0001) (Figure 4B). A similarly strong correlation was seen in recent-onset, mostly untreated RA patients (third-tertile geometric mean (95% CI) 74.1 IU/ml (51.4 to 106.7), first- and second-tertile geometric mean (95% CI) 20.4 IU/ml (15.1 to 27.5); *P* < 0.0001) (Figure 4D). Thus, serum CXCL13 levels exhibited strong correlations with both IgM and IgA RF titers.

## Discussion

We report a strong relationship between elevated serum CXCL13 levels and seropositive RA that was seen in an established disease cohort and confirmed in a mostly untreated early RA cohort. Modest associations were seen with disease activity measures in established RA, but no associations were present in patients with early disease. We did not find any evidence to support a relationship between CXCL13 and HLA-DRB1 alleles containing the shared epitope or complement *C4B* deficiency. The most striking finding was the strength of the relationship between serum CXCL13 with IgM and IgA RF relative to that seen with ACPA titers (Figures 1, 3 and 4), an observation not due to the heterophilic effects of RF. Although an association between CXCL13 and ACPA was observed, it was not as strong a relationship as that seen with RF (IgM RF: *r* = 0.45 and *r* = 0.54 in the established and early RA cohorts, respectively; IgG ACPA: *r* = 0.17 and *r* = 0.21, respectively). These observations suggest interesting and potentially specific associations of CXCL13 with both RF autoantibody formation and the pathogenesis of RA.

We evaluated patients with very high CXCL13 values (top decile) and did not observe any significant variation in RF or ACPA values compared with the remaining patients in the highest tertile (data not shown). Further, we did not identify any competing diagnosis (for example, lymphoma or cryoglobulinemia) or therapy to account for the very high levels of CXCL13.

The presence of elevated CXCL13 levels in seropositive patients from two distinct cohorts composed of patients with established and recent-onset RA suggests that this abnormality may be maintained over the course of the disease and may not be extensively influenced by disease duration or prior therapy. Supporting this notion is our observation that the mean CXCL13 levels in the two cohorts were nearly identical (330 vs 323 pg/ml). By extension, this result would predict the absence of a correlation between serum CXCL13 and disease activity measures. Indeed, only a modest relationship with DAS28-CRP, and no relationship with hsCRP or CDAI, was seen in the Dartmouth RA Cohort. In addition, no relationship was observed with baseline hsCRP, DAS28-CRP or radiographic erosions in the recent-onset RA patients from the Sherbrooke EUPA Cohort (Figure 2A to 2D). Similar findings (modest correlation with DAS28-CRP and no correlation with erosive disease) were recently reported in a French cross-sectional cohort [16]. Thus, elevated serum CXCL13 levels did not simply reflect quantitative differences in synovial or systemic inflammation between patients; rather, these data suggest the presence of a qualitatively distinct subset of seropositive RA manifested by specific increases in both IgM and IgA RF.

This interpretation is further supported by the findings reported in a small study (*n* = 20) demonstrating that serum CXCL13 levels did not correlate with DAS28 measures [11]. Perhaps more important, the same study identified a strong relationship between serum CXCL13 protein and synovial CXCL13 mRNA expression [11]. Thus, serum CXCL13 levels appear to derive from a synovial inflammation process characterized by the production of CXCL13. Although this interpretation differs somewhat from prior reports, these latter studies may have been confounded by inclusion of seronegative patients or the inappropriate use of RF level as a criterion for disease activity [11,17,18]. Clearly, additional studies are needed to clarify this issue.

Murine models indicate that the function of CXCL13–CXCR5 interactions promotes recruitment of B cells and follicular T helper cells to the follicle and germinal centers in secondary lymphoid organs [19]. In humans, the precise role of CXCL13, let alone rheumatoid synovium, is less clear. Synovial expression of CXCL13 has been associated with diffuse lymphoid infiltration as well as the presence of lymphoid aggregates that resemble germinal centers [7,9,20]. However, the relationship of synovial histology to either inflammation or autoantibody production remains controversial [3,21,22].

The surprisingly strong correlation between serum CXCL13 levels and RF (IgM and IgA) titers, relative to that seen with either serum IgG or IgG-ACPA levels, in both an established RA cohort and a recent-onset, mostly untreated RA cohort may clarify the role of CXCL13 in autoantibody production. A similar but lesser relationship was observed in a recent cross-sectional analysis [16]. The most straightforward interpretation is that a greater proportion of circulating RF derives from synovial production relative to IgG-ACPA and IgG, which are presumably produced at other sites, including the bone marrow. Despite its simplicity, this model must account for why both IgM RF and its class-switched counterpart, IgA RF, are affected. Alternatively, high levels of CXCL13 production may reflect a pathologic process in which synovial plasma cell production of RF is selectively enhanced relative to ACPA or other IgGs. Indeed, a strong CXCL13–RF relationship does not establish causality; therefore, another possibility is that elevated RF levels somehow drive increased production of CXCL13.

## Conclusion

In our present report, we demonstrate that serum levels of the B-cell chemokine CXCL13 exhibit a strong relationship with seropositive RA. The nature of this correlation appears to be particularly strong for both IgM and IgA RF, whereas there is a weaker relationship with IgG-ACPA. A particular strength of this finding is its presence to nearly identical degrees in both an early RA cohort and an established RA cohort. Elevations of serum CXCL13 did not consistently associate with disease duration, sex or measures of disease activity in seropositive RA patients. Moreover, CXCL13 levels did not appear to associate with other features of seropositivity, such as the shared epitope. These results suggest that elevated CXCL13 levels may possibly be used to identify a distinct subset of seropositive RA patients that may either promote or result from the expansion of RF-producing B cells.

## Abbreviations

ACPA: Anticitrullinated peptide/protein antibody; CDAI: Clinical Disease Activity Index; CXCL13: C-X-C motif chemokine 13; CXCR5: C-X-C chemokine receptor type 5; DAS28–CRP: Disease Activity Score in 28 joints–C-reactive protein; EUPA: Early Undifferentiated Polyarthritis Cohort; hsCRP: High-sensitivity C-reactive protein; Log CXCL13: Log-transformed C-X-C motif chemokine 13; RA: Rheumatoid arthritis; RF: Rheumatoid factor.

## Competing interests

The authors declare that they have no competing interests.

## Authors’ contributions

JDJ was responsible for the study design, data collection and analysis and drafting and critical revision of the manuscript. BJH performed data collection and analysis and critical revision of the manuscript. GJC carried out data analysis, specimen collection and critical revision of the manuscript. AJF, PC, PL, AM and HM were responsible for patient recruitment and study design and critical revision of the manuscript. NC performed data collection and analysis and critical revision of the manuscript. DLB was responsible for the study design and critical revision of the manuscript. SR was responsible for the study design and performed data analysis and critical revision of the manuscript. GB was responsible for the study design, performed data collection and analysis and drafted and critically revised the manuscript. WFCR was responsible for the study design, performed data collection and analysis and drafted and critically revised the manuscript. All authors read and approved the final manuscript.

## Supplementary Material

Additional file 1: Figure S1 Evaluation of combined data from seropositive patients from the Dartmouth and Sherbrooke cohorts. The evaluation did not identify any relationship with shared epitope status or smoking. **(A)** Log C-X-C motif chemokine 13 (CXCL13) levels do not vary based on the presence or absence of the shared epitope (*n* = 258, *P* = 0.73). **(B)** Log CXCL13 levels have no relationship with smoking whether comparing current smokers (*n* = 80) to past smokers (*n* = 129; *P* = 0.69), current smokers to never-smokers (*n* = 125; *P* = 0.28) or current smokers to both past and never-smokers (*P* = 0.42). Click here for file
