# Supplementary material for: Serum C-X-C motif chemokine 13 is elevated in early and established rheumatoid arthritis and correlates with rheumatoid factor levels
Source: Arthritis Res Ther. 2014 Apr 25;16(2):R103. doi: 10.1186/ar4552 (PMC4060390; doi:10.1186/ar4552)
Supplement: Additional file 1: Figure S1 — Evaluation of combined data from seropositive patients from the Dartmouth and Sherbrooke cohorts. The evaluation did not identify any relationship with shared epitope status or smoking. (A) Log C-X-C motif chemokine 13 (CXCL13) levels do not vary based on the presence or absence of the shared epitope (n = 258, P = 0.73). (B) Log CXCL13 levels have no relationship with smoking whether comparing current smokers (n = 80) to past smokers (n = 129; P = 0.69), current smokers to never-smokers (n = 125; P = 0.28) or current smokers to both past and never-smokers (P = 0.42). [file ar4552-S1.pdf]

Additional Figure 1

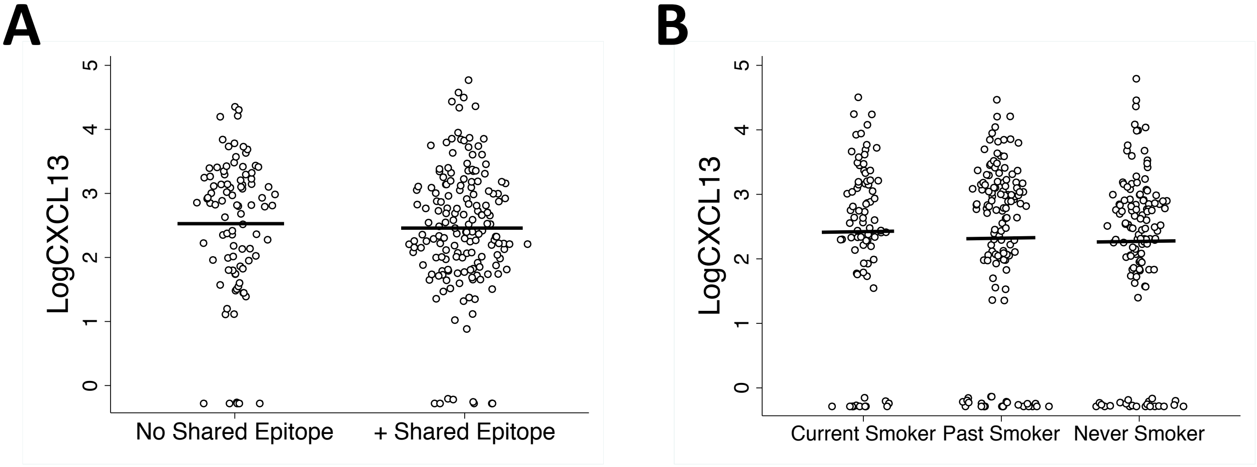

Additional Figure 1. Evaluation of combined data from seropositive patients from the Dartmouth and Sherbrooke Cohorts does not identify any relationship with shared epitope status or smoking. **A,** LogCXCL13 levels do not vary based on the presence or absence of the shared epitope (n=258, p=0.73). **B,** LogCXCL13 levels have no relationship with smoking whether comparing current smokers (n=80) to past smokers (n=129, p=0.69), current smokers to never smokers (n=125, p=0.28), or current smokers to both past and never smokers (p=0.42).
